# The Progress of Pancreatectomy for Pancreatic Cancer Treatment—Lessons Learned and Future Challenges

**DOI:** 10.3390/cancers18142297

**Published:** 2026-07-16

**Authors:** Reinhold Függer, Matthias Biebl

**Affiliations:** 1 Department of General, Visceral, Thoracic and Transplant Surgery, Ordensklinikum Linz, 4020 Linz, Austria; matthias.biebl@ordensklinikum.at; 2 Faculty of Medicine, Johannes Kepler University, 4040 Linz, Austria

**Keywords:** pancreatic resection, pancreatic ductal adenocarcinoma, mortality and morbidity, resectability, multimodal therapy, tumor biology

## Abstract

Since decades, surgery of pancreatic adenocarcinoma is confronted with two major challenges. First, the prognosis of pancreatic adenocarcinoma is the worst of all gastrointestinal malignancies, characterized by late diagnosis and aggressive tumor biology. Second, postoperative mortality and morbidity have been exceptionally high, giving pancreatic resection a reputation of being one of the most dangerous procedures. This narrative review concentrates on the progress in decreasing postoperative mortality by centralization and standardization, the exertion of extended resections for local tumor clearance and multimodal strategies to improve oncologic survival. Research regarding surgical techniques, especially robotic assistance, indicated further progress in minimizing the perioperative trauma. Multimodal treatment concepts have been implemented with adjuvant therapy in resectable and neoadjuvant regimen in borderline and locally advanced cancer. However, significant problems have to be solved. Postoperative morbidity remains high, hindering the administration of adjuvant therapy, and toxicity is an essential factor in neoadjuvant strategies. Despite neoadjuvant therapies, resection rates of locally advanced carcinoma are modest and tumor progression hinders resection of borderline and resectable carcinomas. Obviously, deficits in understanding tumor biology are a central obstacle in improving resection rates and overall survival. Hence, improvement in selection of patients for surgical resection apart from using preoperative anatomical findings is mandatory. The progression of current standards, future developments and the status of surgery in multimodal concepts to treat pancreatic adenocarcinoma are delineated using historical and recent reports, registry data, meta-analysis and randomized controlled trials.

## Simple Summary

Pancreatic cancer represents the fourth leading cause of cancer-related death, and is expected to rise to the second due to increasing incidence. It is characterized by late diagnosis and aggressive tumor biology. Only 20% of patients diagnosed with pancreatic cancer are candidates for potentially curative treatment consisting of surgical resection and multimodal therapy. This narrative review summarizes the progress achieved by centralization of pancreatic surgery, minimizing the operative trauma and implementation of multimodal treatment concepts. Future challenges to improving resection rates and survival are addressed. Recent data favor a personalized treatment strategy based on cancer anatomy, tumor biology and condition to select patients for curative surgery embedded in a multimodal concept. Concrete questions to solve are the role of neoadjuvant therapy in primarily resectable carcinoma, patient selection for surgery of borderline resectable and locally advanced cancer and proper assessment of tumor biology.

## 1. Introduction

Pancreatic adenocarcinoma (PDAC) remains one of the most fatal gastrointestinal malignancies. Its incidence is increasing steadily and it represents the fourth leading cause of cancer-related death, and is expected to rise to the second in international and national estimations by 2030 [1].

Surgical resection is a prerequisite for cure in a multimodal treatment concept. A pivotal problem of treatment strategies for PDAC is that only 15–20% are resectable at the time of diagnosis. According to a worldwide registry-based analysis of trends in cancer survival for 2000–2014, this results in 5-year-survival rate of only 5–15% [2]. Surveys of long-term survival in resected patients undergoing multimodal neoadjuvant or adjuvant therapy reported 5-year-survival rate of 27% in a cohort treated between 1990 and 2002 [3]. Another historical series with upfront resection and adjuvant therapy attained an overall 18.8% 5-year-survival rate in the period of 2001–2011 and 40–58% in favorable subgroups of Stage IA and IB [4]. Although comparison of reported series is limited by divergent patient selection, surgical standards and oncologic therapy, these data represent a modest improvement of outcome. However, pancreatic cancer remains to be the deadliest cancer of the gastrointestinal tract.

Historically, from the beginning of consideration of treatment strategies, pancreatic resection has been at the center of debates, reflecting the area of conflict between the prerequisite of surgery for curative therapy and the burden of surgical complications and mortality. This review spans a bridge from standardization of surgery for PDAC to improving postoperative mortality by centralization, reducing morbidity by minimally invasive techniques and to the role of NAT in today’s multimodal concepts. Finally, a clinical pathway of decision-making and patient selection is provided, including an outlook on recent research exploring tumor biology, that may help to improve results apart from specific surgical tasks.

## 2. Methods

We searched the databases PubMed, Embase and Cochrane with the primary keywords pancreatic ductal adenocarcinoma and pancreatic resection. Secondary keywords were postoperative mortality, caseload, centralisation, adjuvant therapy, neoadjuvant therapy, and surgical technique. References include registry data, RCTs, meta-analyses and large multi- and single-center reports including historical landmark publications. As this is a narrative and not a systematic review, the selection of the literature was chosen subjectively.

## 3. Postoperative Mortality and Centralization

In the 1970s and 1980s mortality rates associated with pancreatic surgery were extremely high. Bramhall et al. reported a resection rate of 2.6% with a mortality of 27.6% in an epidemiological study of pancreatic adenocarcinoma in the West Midlands [5]. It is not surprising that pancreatic resection had an exceptionally poor reputation and even the basis for surgery as part of the therapeutic concept in patients with resectable pancreatic carcinoma was challenged [6]. Consequently, resection rates were low. In contrast to such dismal reports, there was notice of another way. Surveys including up to 1000 resections were published by Johns Hopkins for the 1970s–1990s, indicating an in-hospital mortality of around 1% and Warshaw-published resection rates of 5–10% resulting in median survival rates of 17–20 months [7,8,9]. First notes of an advantageous correlation of caseload and surgical mortality were published. A survey of specialist pancreatic units from the UK, encompassing 1026 pancreatic resections from 1990 to 1996, reported a median case load of 5.5 pancreatoduodenectomies per year compared to 0.5 in general units. Concurrently, they found a significant difference in mortality rates of 4.9% in specialist versus 9.8% in general departments [10]. Hence, the volume–mortality relationship was a dominating subject in outcome research. National surveys reporting postoperative mortality and the correlation with the yearly numbers of resections made pancreatic surgery the most investigated surgical procedure. Birkmeyer et al. published their landmark study on hospital volume and surgical mortality in 2002, reporting a postoperative mortality of 14.6% in departments performing 1–2 resections compared to 3.8% in units with more than 16 resections [11]. Consequently, a plea for centralization was brought forward, resulting in minimal caseloads prescribed by national health authorities. One leading country regarding the centralization of pancreatic surgery is the Netherlands. Between 2004 and 2009, surgical units providing pancreatic resections were reduced from 48 to 30 and hospital mortality decreased from 9.8% to 5.1% [12]. National data from the United States, summarizing 279,445 patients with primary pancreatic neoplasm between 1998 and 2003, revealed a resection rate of 14% and an overall in-hospital mortality of 5.9%, with a significant decrease from 7.8% to 4.6% [13].

Although the decrease in post-pancreatectomy mortality and its relation to volume could be clearly deduced from numerous publications in the 2000s, surprisingly high mortality rates were published even a decade later. An analysis of pancreatic resections, using a database of 58,003 resections between 2009 and 2013, showed an overall mortality of 10.1%, which did not change during the study period [14]. In malignant pancreatic tumors, in-hospital mortality was 5.1% for distal pancreatectomy, 6.3% for pylorus-preserving pancreatoduodenectomy, 9.5% for Whipple and even 19.9% for total pancreatectomy. Looking for an explanation of high mortality rates, inconsequent centralization by setting low national minimal caseloads and hospitals continuing to perform pancreatic resections with low volume may explain the high mortality rates. A survey on the mortality rate in all Austrian hospitals performing pancreatic resections revealed a median number of 10.5 resections per hospital. This is remarkable, because a minimum caseload of 10 resections per year was preset by the health authorities. Nearly half of pancreatic resections performed in hospitals were below the preset volume. The in-hospital mortality in units with less than 10 resections was 12%, compared to 5.8% in hospitals with a minimum of 30 resections [15].

A possible bias in the interpretation of postoperative mortality rates may be that most data come from administrative databases and only few come from clinically reviewed registries. While clinical registries offer detailed scientific insights, they are restricted to participating centers and often lack completeness from the national point of view. On the other hand, administrative data are able to deliver a complete national overview, but are not appropriate for analyzing more complex clinical questions. Table 1 summarizes national and registry data on mortality following pancreatic resection.

Superior results of high-volume institutions have been published regarding post-resectional margin status and long-term survival [17,18]. Furthermore, superior results in complication management and lower rates of failure to rescue are associated with higher caseloads [19]. This indicates also a shift in quality assessment from pure perioperative mortality rates to more differentiating quality parameters like clinically relevant morbidity or failure to rescue. Finally, surgical education in pancreatic surgery presumes a high volume of resections. Tseng et al. determined the learning curve of pancreatoduodenectomy to be as high as 60 procedures when analyzing improvement across the parameters of estimated blood loss, operative time, length of stay and rate of microscopically positive resection margin [20].

## 4. Reducing Surgical Trauma and Enhancing Recovery

First reports of laparoscopic distal pancreatectomy and pylorus preserving pancreatoduodenectomy were published in the 90s in the early phase of laparoscopic gastrointestinal surgery [21,22]. Patient numbers were very low and pancreatic resections remained pretty rare. In contrast to pancreatoduodenectomy, laparoscopic distal pancreatectomy achieved increasing acceptance in the 2000s and 2010s for benign and malignant indications. Regarding PDAC, short- and long-term oncologic outcomes were not different between laparoscopic and open distal pancreatectomy. Due to a reduction in hospital stay and operative trauma, the laparoscopic approach was preferred increasingly, whenever it was judged feasible [23]. Subsequently, a nationwide RCT from The Netherlands resulted in better functional recovery and a reduction in intraoperative blood loss following minimally invasive distal pancreatectomy, and no differences in the overall rate of postoperative complications [24]. Regarding laparoscopic pancreatoduodenectomy, trials brought up conflicting results on postoperative complications initially. One RCT (LEOPARD-2) was interrupted prematurely, because of a higher incidence of complication-related deaths in the laparoscopic group compared to the open group (10% vs. 2%, RR 4,9; *p* = 0.2). However, LEOPARD-2 resulted in important learnings regarding effects of learning curves, surgeon credentialing and center selection. Thus, this trial may be seen more as a step towards optimally planning a trial in complex, minimally invasive surgery than a failure of laparoscopic pancreatoduodenectomy [25].

With the spread of robotic assistance, minimally invasive pancreatoduodenectomy is performed increasingly and two RCTs stated its safety and non-inferiority regarding procedure-specific complications compared to open pancreatoduodenectomy [26,27]. Both RCTs found a reduction in intraoperative blood loss, shorter hospital stays and earlier rehabilitation. Limited by their small sample size, these RCTs do not allow general recommendations, but underline the need for larger multi-centric trials.

Searching for reasons behind the striking spread of robotic pancreatoduodenectomy, the alleviation of surgical reconstruction seems to be the most important driver [28]. The set-up of benchmarks to develop a standard for robotic resections is a next step to compare outcome quality [29].

It has been shown that extended resections including vascular resections and reconstructions are also feasible [30]. However, large data on extended robotic pancreatectomy providing information on safety, applicability, potential limits and advantages compared to open surgery are still missing.

Minimizing the operative trauma and decreasing postoperative recovery is not solely driven by technical progress and increasing experience in surgery, but is also generally supported by a change in postoperative care to evidence-based concepts. ERAS challenged traditional positions regarding the use of postoperative drains, gastrointestinal tubes, nutrition, and remobilization and resulted in a significant decrease in the length of hospital stays and rehabilitation periods [31]. Thus, the concept of ERAS is an integral part of efforts to minimize operative trauma, reduce postoperative morbidity and enhance recovery.

## 5. Postoperative Morbidity and Clinical Consequences

While postoperative mortality decreased significantly, morbidity could not be improved by a comparable amount. Overall postoperative morbidity following pancreatoduodenectomy is reported in a range of 40–50%; severe complications rated according to Clavien-Dindo III and higher occur in up to 25% [32].

The most feared complication associated with postoperative mortality is the development of postoperative pancreatic fistulas (POPF). This has not changed over time. However, a generally accepted definition of POPF and a grading according to clinical consequences have been published by the International Study Group of Pancreatic Fistula (ISGPF) and revised accordingly [33]. While a biochemical leak is defined as an elevated level of amylase in the drained fluid without clinical signs of infection or deterioration of the patient’s course, CR-POPF are differentiated into Grade B and C according to their clinical relevance. The differentiation between a biochemical leak and clinically relevant pancreatic fistulas (CR-POPF) was a big step forward in the understanding of the pathophysiology of POPF and the development of an adequate, goal-oriented complication management. Nevertheless, CR-POPF is the most prominent severe complication following pancreatoduodenectomy and responsible for 57% of deaths within 60 days past surgery [34]. CR-POPF is the dominant cause of unplanned reoperations and reinterventions, which in turn are associated with increased mortality [35].

Complication management changed since the 2000s markedly. While the rate of relaparotomies decreased, percutaneous CT or ultrasound-guided drainages of fluid collections and intra-abdominal abscesses were used increasingly. Endosonographic-guided insertion of lumen-apposing metal stents is applied to evacuate well-confined retentions and detritus in the later course of CR-POPF [36]. Angiographic stenting and/or coiling replaced surgical revision whenever judged appropriate in CR-POPF-related post-pancreatectomy hemorrhage [37].

The need for relaparotomy, once the cornerstone of surgical complication management, decreased over the past decade due to these interventional techniques. Most indications for relaparotomy are seen in the very-early postoperative period, in case of early postoperative hemorrhage, insufficient organ perfusion or occlusion of vascular reconstructions. There is also a tendency for pancreas-preserving revisional surgery instead of completion pancreatectomy for surgical management of POPF Grade C [38]. It is a fact that the role of relaparotomy in treatment strategies of CR-POPF has changed from a must to a policy that could be summarized as “more is not necessarily better”.

A major issue addressed in the recent literature is the consequence of postoperative morbidity, especially of CR-POPF on the delivery of adjuvant chemotherapy. About 29% to 33.9% of patients miss subsequent adjuvant chemotherapy after resection of PDAC, which is of negative impact on overall survival [39]. According to data from the National Cancer Database including 13,438 patients operated on from 2006 to 2015, improvement of survival by adjuvant therapy was independent of a potential delay. A benefit was seen in patients starting adjuvant therapy < 6 weeks, 6–12 weeks and 12–24 weeks after PDAC resection. CR-POPF and postoperative bile leakage are significant causes of omission of adjuvant therapy and survival is decreased by POPF Grade C compared to patients with no POPF (20.22 vs. 26.33 months) and to those with POPF Grade B (20.22 vs. 26.87 months). In particular, Grade C patients who received adjuvant chemotherapy in only 26.7% vs. 74.9% in all other patients, were negatively affected [40,41,42].

Minimizing the operative trauma by robotic pancreatectomy has an impact on the receipt of adjuvant chemotherapy. In a matched cohort study, 96.2% of patients after robotic pancreatectomy and 78.9% after open pancreatoduodenectomy received chemotherapy. In the robotic group, 73.1% returned to their intended oncologic therapy within 8 weeks compared to 44.2% in the open group [43]. Complex upfront resections like pancreatectomies with venous resection and reconstruction carry a particular risk of missing adjuvant therapy in the case of major complications. Of the patients, 30% missed adjuvant therapy due to complications with a median OS of 10 months vs. 36 months in those receiving adjuvant therapy [44]. The impossibility of administering adjuvant therapy due to severe postoperative complications is an argument for expanding the use of neoadjuvant therapy (NAT) to anatomically resectable patients.

Interestingly, the rate of CR-POPF was lower in patients scheduled for neoadjuvant therapy and subsequent resection compared to upfront surgery (3.8% vs. 13.8%, *p* = 0.001) without differences in severe complications of other origins and in mortality. Although the pathophysiologic mechanism behind this observation is not clear yet, it may be speculated that the induction of fibrosis by NAT may play a role [45,46].

Infectious complications are of particular importance regarding postoperative morbidity and mortality [47]. Surprisingly, the literature on the impact of infectious complications following pancreatoduodenectomy is scarce. Kachfe HH et al. published a 30% rate of infectious complications. With respect to OS, they report a median of 33.3 months, 29.06 months and 27.58 months (*p* = 0.049) for patients experiencing no complication, noninfectious complications and infectious complications, respectively [48].

## 6. Surgical Technique—Standard and Extended Pancreatectomies

Over the decades, the optimal technique for performance of pancreatic resections was a central subject of research and publications. Research topics concentrating on surgical details included Whipple vs. pylorus-preserving pancreatoduodenectomy, reconstruction by pancreatojejunostomy vs. pancreatogastrostomy, numerous more or less varying techniques for pancreatojejunal anastomosis, the use of external or internal transanastomotic stents in pancreatojejunostomy, surgical methods for optimal handling of the pancreatic stump in distal pancreatectomy, advantages or drawbacks of resecting the pyloric ring in pancreatoduodenectomy, to name the most prominent [49,50,51]. This list could be continued by items concerning preoperative care like fistula prophylaxis by reducing pancreatic fluid secretion, the usage of gastrointestinal tubes, and several methods of infection prophylaxis, without being able to really mention all surgical and preoperative details meant to be essential for a successful pancreatectomy. An example for current clinically oriented surgical research may be the scientific question on the use of routine abdominal drainage. Given a historically based policy of an absolute demand to insert prophylactic drains, the routine has changed during the past 15 years. According to several RCT, a recent meta-analysis provides high evidence for selective early drain removal in pancreatoduodenectomy and omission of routine drainage in distal pancreatectomy [52].

Today, controversies on these partly emotionally boosted questions have calmed down and a pragmatic approach dominates most of the topics. More philosophically, one could note that there is not only one path to success in pancreatic surgery. The assessment of quality in pancreatic surgery is based on objective outcome parameters rather than adherence to specific surgical techniques.

In a consensus statement of the ISGPS, a definition of standard lymphadenectomy was published [53]. For pancreatoduodenectomy standard lymphadenectomy includes lymph node stations 5, 6, 8a, 12b1, 12b2, 12c, 13a, 13b, 14a, 17a and 17b (Japanese classification). In distal pancreatectomy for cancers located in the body and tail, stations 10, 11, and 18 are removed. Although no survival benefit was demonstrated in former publications, there is an ongoing controversy on the extension of lymphadenectomy. The reason for considerations of extended clearance of lymphatic tissue is that most local recurrences are located in relation to the SMA. Consequently, the origins of the SMA and the mesopancreas are of particular interest for surgery. Several variations to clear this triangle by extended open or robotic pancreatoduodenectomies have been described [54,55]. Extended TRIANGLE resections resulted in higher numbers of lymph nodes harvested and a decrease in R1 rates compared with standard pancreatoduodenectomies. Lei et al. published a significant delay in tumor progression in patients with extended lymphadenectomy based on the TRIANGLE operation [56]. Recently David et al. reported mesopancreatic infiltration in 76.9% of patients with upfront surgery of primarily and borderline resectable PDAC. Mesopancreatic infiltration correlated significantly with incomplete resection. This high rate of mesopancreatic tumor spread raises questions concerning resectability classification, standard pancreatoduodenectomy and NAT for patients classified as resectable [57].

Although it is evident that an extension of pancreatic resection to vessels and adjacent organs has an impact on the surgical challenge and outcome, it took time for a standardized approach to be developed. Based on 3953 pancreatoduodenectomies performed between 2001 and 2019, four types of pancreatoduodenectomy were proposed according to surgical difficulty and extension by Mihaljevic et al. [58]. They differentiated between standard pancreatoduodenectomy in 74.1% (Type 1), pancreatoduodenectomy with portal/superior mesenteric vein resection in 14.4% (Type 2), pancreatoduodenectomy with multivisceral resection in 10.3% (Type 3) and pancreatoduodenectomy with arterial resection in 1.0% (Type 4). These resection types differ significantly in postoperative mortality with 2.9%, 4.2%, 6.3% and 10.3% for types 1–4, respectively. Differences are observed in postoperative morbidity, CR-POPF, delayed gastric emptying and rates of relaparotomy. While it is not surprising that surgical complexity has been increasing in the current era of neoadjuvant therapy and efforts to convert locally un-resectable pancreatic cancer, a trend to extended resections has been evident for the last few decades. In a survey of all pancreatoduodenectomies between 1988 and 2020, an increase in surgical difficulty (defined type 2–4 pancreatoduodenectomies) from 6.1% to 14.6% and 22.0% was found for the periods 1988–2000, 2001–2010 and 2011–2020, respectively [59]. In the same periods, 30-day mortality rates decreased from 6.4 to 5.7 and 2.9%. By 2035 extrapolation of this data revealed a continuing increase in complexity with a rate of 50% for vein resection. Apart from these high rates of portal vein resection in very-high-volume centers, there is a trend to extend pancreatoduodenectomy to vein resection since the beginning of the 2000s. Data from the Nationwide Inpatient Sample showed an increase from 0.7% to 6.0% between 2000 and 2009 [60].

Pancreatoduodenectomies with vein resection are not only associated with elevated postoperative mortality, but also with higher rates of R1/R2 resection and a significantly shorter overall survival compared to those without vein resection [61]. Historically, these impaired results have been interpreted as sequelae of higher technical difficulty. Without doubt, perioperative results are affected by technical issues, but more likely the impaired overall survival refers to an advanced disease as an underlying cause in explaining the worse survival after vein resection. In a survey from Heidelberg 30.6% of procedures included vein resections, with a 90-day mortality of 6.4% and median overall survival of 23.3 months in R0 resections [62]. Albeit worse than survival of patients undergoing standard pancreatoduodenectomy for PDAC, this remains superior to outcomes without resection. The ISGPS published a categorization of portal vein resection and reconstruction depending on the extent of resection and reconstruction techniques [63]. Resection and end-to-end reconstruction is the most applied technique. Thrombosis of the reconstruction was reported in 21.2% and associated with impaired survival, especially for reconstructions with interposition grafts.

Superior mesenteric vein involvement in locally advanced pancreatic cancer is a particularly demanding situation for resection. Apart from intraoperative confirmation of resectability and defining appropriate vascular anatomy for reconstruction, portal hypertension and portomesenteric collateralization aggravate surgery markedly. According to the individual situational preservation of collaterals, mesoportal preresectional bypass, mesoportal interposition graft and mesocaval shunt are appropriate surgical techniques to handle this challenge.

Regarding the high mortality of arterial reconstructions, periadventitial divestment gained some interest. While this technique reduces risks associated with arterial resection and reconstruction, limits in achieving R0 resection dependent upon the extent of circumferential and radial tumor infiltration confine periadventitial divestment to individual application. Butler et al. reported in their meta-analysis R1 resection rates of 16–71% and a positive margin of the SMA in 15–45% [64].

Distal pancreatectomy with coeliac axis resection represents a specific form of extended resections. Originally described by Appleby for the resection of advanced gastric cancer with coeliac axis involvement, the procedure enables the resection of pancreatic cancer of the corpus pancreatis with local infiltration of the coeliac axis using the route of the superior mesenteric and gastroduodenal artery to preserve the hepatic arterial blood supply after coeliac axis resection [65]. Case numbers are limited, but retrospective surveys from the Mayo Clinic and Johns Hopkins indicate a distinct improvement with postoperative mortality of 2–4%, R0 resection in 88% and overall survival of 25 and 36.2 months [66,67]. Due to the complex vascular challenge and high morbidity this procedure should be limited to fit patients with response to neoadjuvant therapy in specialized centers.

## 7. Causes of Mortality

In contrast to mortality rates, that were at the center of concern since decades, causes of mortality are a rare subject of outcome research. Recently, the ISGPS published a definition and classification of post-pancreatectomy mortality [68]. Causes of death were classified according to the categories vascular/technical (Post-Pancreatectomy Mortality 1—PPM 1), post-pancreatectomy-specific complication-related mortality (PPM 2) and cardiopulmonary/cardiovascular mortality (PPM 3), with the literature-derived ranges of 15–30%, 45–65% and 10–25%, respectively. This categorization summarizes what can be extracted from numerous national and institutional surveys on the morbidity and mortality of pancreatic surgery. In particular, pancreatectomy-specific complications may lead to systemic organ dysfunction, finally causing death. The most prominent example is the development of postoperative pancreatic fistula, that represents the most frequent lethal complication of pancreatectomy. Following the trend to extended resections for locally advanced pancreatic adenocarcinoma and the accompanying need of vascular resections and reconstructions, mortality related to technical and vascular complications will be under close observation.

With respect to pancreatectomy-specific complications, 57% of deaths have to be attributed to CR-POPF. Concurrently, a remarkable delay in diagnosis and therapeutic escalation was reported in this recent survey [34]. In the context of surgery-specific complications, it has to be underlined that infection is of decisive importance for deteriorating the clinical course of patients with CR-POPF. Mostly, POPF is estimated to be sterile in the beginning. Depending on time, infection will develop and may cause lethal septic organ failure. Early diagnosis and source control are crucial in the management of post-pancreatectomy complications.

## 8. The Impact of Neoadjuvant Therapy on Resectability and Survival

In the concept of multimodal treatment strategies in potentially curative patients with PDAC, the goals pursued by neoadjuvant therapy are to increase resectability and survival. Originally resectability was defined by local anatomy and the absence of distant metastasis according to preoperative staging. Differentiation of anatomy in primarily resectable, borderline resectable and locally advanced PDAC, assessment of tumor biology according to CA 19-9 levels and patient condition allowed a more complex estimation of resectability and promoted progression in multimodal strategies by defining patient subgroups [69,70].

An improved survival rate following the conception of NAT and resection in borderline resectable and locally advanced PDAC is published in RCT, meta-analysis and selected series [71]. In a recently published multicenter study of patients with locally advanced PDAC and induction therapy with FOLFIRINOX, CA 19-9 decrease >80%, post-induction CA 19-9 < 50 mL/U and WHO performance status 0 achieved the best three-year survival rate, thus strengthening the ABC concept in patient selection for surgical resection [72]. In a similar multicenter analysis in localized PDAC with neoadjuvant FOLFIRINOX tumor anatomy (borderline resectable and locally advanced), CA 19-9 > 500 U/mL and performance status ≥ 1 were identified as prognostic factors. A score calculated from these factors resulted in 5-year overall survival rates of 47%, 28.9%, 19.2% and 4.8% [73].

The role of NAT in resectable pancreatic cancer is at the center of ongoing debates. Results of meta-analyses and RCTs were not consistent regarding overall survival, when comparing NAT and upfront surgery [74,75,76,77,78]. This may be explained by shortcomings in patient selection resulting in inadequate trial design. In this context consideration of tumor biology and drug toxicity are major items that need closer attention.

According to the ABC classification, tumor biology is predominantly assessed by CA 19-9 levels. CA 19-9 is generally available and there is experience regarding levels at different phases during the course of PDAC, such as diagnosis, treatment response and surveillance. However, CA 19-9 is limited by non-secretion in about 10% of patients and by interaction with bilirubin levels. The use of ctDNA may be an option for a more appropriate biologic staging and monitoring of the therapeutic response, but has not passed into clinical praxis widely [79]. Another approach to assessing tumor biology may be upfront molecular profiling as used in the COMPASS Trial for a personalized treatment regimen in anatomically resectable, borderline or locally advanced patients with PDAC [80]. Hope for future progress by therapeutic interventions may be derived from a retrospective analysis of the molecular profile of 342 patients with PDAC, where actionable alterations were identified in 20% [81]. In this context, the limited response rates of the currently used chemotherapy regimens folfirinox and gemcitabine/nab-paclitaxel are underrepresented in the ongoing discussion [82].

A meta-analysis by Brown et al., including 125 studies and 11,713 patients undergoing neoadjuvant chemotherapy, chemoradiation or chemotherapy and radiation, revealed resection rates of 77.4, 60.6 and 22.2% for preoperatively resectable, borderline resectable and locally advanced classified patients, respectively [83]. Overall survival for resected patients was superior compared with non-resected patients, with 38.5 vs. 13.3, 32.3 vs. 13.9 and 30.0 vs. 14.6 months for resectable, borderline resectable and locally advanced PDAC, respectively. Although these data demonstrate a significant survival benefit for surgical resection, resection rates of primarily resectable PDAC demand further reflection. A resectability rate of 77.4% in patients preoperatively classified as resectable and undergoing NAT is a modest result. These patients are submitted to neoadjuvant treatment mainly to optimize their systemic oncologic situation and not for local downstaging to enable resection. The conclusion is that nearly a quarter of patients staged with resectable PDAC develop progressive disease during NAT. This progress may be explained by non-resonance to preoperative therapy or deficient rating of tumor biology. Concrete data on the reasons for not undergoing resection after NAT are scarce. For patients classified as resectable PDAC, Brown et al. report distant progression in 8.7%, occult metastasis in 6.8% toxicity in 6.6% and local progression as the most frequently observed causes. Results for borderline resectable PDAC are similar with 12.7% distant progression, 10.3% local progression or un-resectability and 7.4% toxicity. On the other hand, in patients with locally advanced PDAC the most frequent reason to miss resection is local progression or un-resectability in 23.1%, followed by distant progression in 16.7% and toxicity in 10.2%. Apart from missing surgery after NAT for the reasons mentioned above, very-early recurrence after NAT and subsequent curative resection is another clinical note for strengthening doubts in the understanding of tumor biology and appropriate patient selection. A recent survey published recurrence rates of 9.1% within three months after surgery [84].

In comparison to the meta-analysis of Brown et al., resection rates of 40% following NAT for borderline resectable PDAC and 8% for locally advanced PDAC demonstrate the wide range of results [85,86]. Except differences in treatment regimen and selection bias, surgical policy, specifically the access to vascular resection, may explain divergent resection rates.

In some publications the term conversion surgery is used, meaning pancreatic resection following neoadjuvant therapy [87,88]. However, in the absence of a specific definition, the term describes the clinical scenario of resection of locally advanced PDAC after neoadjuvant therapy.

In view of the trend to NAT in PDAC, the results of upfront surgery and adjuvant chemotherapy have to be noted. Strobel et al. published a series of 937 patients with primarily resectable and borderline resectable PDAC, operated on between 2001 and 2011 [4]. Median survival was 22.1 months and actual 5-year survival rate was 17%. In PDAC Stage IA and IB 5-year survival rate was 58.3 and 40.0%. These results in resectable PDAC with adjuvant regimen, that appear suboptimal in today’s assessment, are remarkable. In the absence of a high level of evidence for NAT in resectable PDAC, this discussion is continued [89]. Resection rates of upfront surgery and following NAT are summarized in Table 2.

## 9. Clinical Pathway

According to the literature discussed before and personal experience, we propose a short summary of a clinical pathway for patients diagnosed with localized PDAC. After complete staging patients should be assessed according to the ABC criteria for anatomy (resectable, borderline resectable and locally advanced carcinoma), tumor biology (CA 19-9) and WHO performance status (ECOG).

Patients with resectable anatomy, CA 19-9 < 500 U/mL and ECOG 0–1 may be candidates for upfront surgery or NAT depending on the center’s policy and patient informed consent. Patients with resectable tumors but CA 19-9 levels > 500 U/mL, independently from ECOG status and all patients with borderline resectable or locally advanced anatomy are candidates for NAT and radiologic restaging including CA 19-9. The literature on judgment of therapeutic response is not completely consistent, but normalization of CA 19-9 or a decrease of more than 80% are estimated to be indicators of successful therapy. The duration of NAT varies from two to six months or 4 to 12 cycles. Restaging is indicated at at least three months or earlier in shorter NAT courses.

Patients with positive response to NAT are candidates for exploration and if possible resection. Depending on the local tumor anatomy, resection and reconstruction of the portal and superior mesenteric vein and if appropriate arterial divestment or resection and reconstruction have to be planned. Coeliac axis resection by the Appleby procedure is an opportunity for carcinomas involving the coeliac trunk. All these extended vascular resections and reconstructions should be performed in very-high-volume centers due to their increased risk of morbidity and mortality.

In patients with a course of NAT shorter than 6 months, multimodal therapy should be continued postoperatively for completion.

## 10. Future Directions and Conclusions

Details of surgical techniques dominated studies and debates on pancreatic surgery over the past decades. The most addressed surgical question concerned the ideal pancreatic anastomosis in pancreatoduodenectomy, but pylorus preservation, the extent of lymphadenectomy, pancreatic stump closure, drain management and many other topics were also dominating issues of research on pancreatic surgery. Guidelines and classifications were derived and pancreatic surgery was standardized in many aspects.

Early recognition and consequent therapy of postoperative complications are persistent cornerstones of successful surgery and indicators of outcome quality. Pancreatic surgery bears a high risk of postoperative complications demanding vigorous management. Infection is especially a subsequent and frequent sequalae of complications that demands increased attention in efforts to further improve mortality and morbidity.

The recent years brought a shift in technique. After some time of adopting and developing, there has been a continuous increase in robotic-assisted resections. This technique allows us to overcome the boundaries of classic laparoscopic pancreatic surgery, especially regarding pancreatoduodenectomy. Without prophecy, it is to be expected that robotic-assisted operations will be the dominating approach in surgery of PDAC. Future development and progress will take place in close connection with further optimization of robotic surgery. This technical evolution continues beyond standard resections and encompasses extended pancreatic surgery [31]. According to recently published data from the NSQIP, minimally invasive pancreatoduodenectomy doubled from 6.3% to 14.7% between 2014 and 2023 [94]. However, learning curves of robotic pancreatic resections are long, which will consequently strengthen further centralization to achieve acceptable perioperative results [95].

Another item for future perspectives is the increasing percentage of extended resections. Vascular resections in pancreatoduodenectomy are especially of concern. While portal or SMV resections make up more than 50% in selected series of pancreatoduodenectomy, arterial resections also increased recently [93]. This is caused by the quest to achieve R0 resections after neoadjuvant treatment in locally advanced PDAC. While postoperative mortality is elevated compared to standard resections, the goal of neoadjuvant pretreatment and arterial resection is to increase resectability rates and to offer potential curative surgery to more patients. Given the modest resection rates, realization of this concept is only possible in a minority of patients. Despite the surgical experience and progress of technical equipment, limited response to neoadjuvant regimen and toxicity affect results of neoadjuvant therapy. However, the most stringent reason for missed resectability may be the lack of clarity in understanding tumor biology and faults in patient selection. Obviously, current neoadjuvant regimens and improved surgical techniques alone may not be able to further enhance resection rates without decisive progress in understanding the biology of PDAC. Most recently, RAS inhibition showed promising results in improving survival in pretreated non-resectable patients [96]. Yet, far from integration in surgical multimodal concepts, it may be seen as progress in targeting genomic mutations in PDAC patients.

Last but not least, the most important challenge in the future of surgical therapy of PDAC is the shift from pure anatomical criteria determining resectability to a personalized concept including anatomy, tumor biology and condition. The ABC classification may be seen as basis for future progress integrating modern surgical technique and multimodal therapy to improve survival.

## Figures and Tables

**Table 1:** National/regional mortality of pancreatic surgery in registries.

Author	Country	Period	Hosp/Pat *n*	Surgery	Mortality %
Neoptolemus (1997) [10]	UK	1976–1996	21/1026	PDAC	5.7
Birkmeyer (2002) [11]	US	1994–1999	1868/10,530	PS	3.8–17.6
McPhee (2007) [13]	US	1998–2003	-/39,463	PD	5.9
De Wilde (2012) [12]	NL	2004–2009	48 → 30/-	PD	9.8 -> 5.1
Nimptsch (2016) [14]	G	2009–2013	22,848/-	PS	8.6
Cardini (2019) [15]	A	2017	60/-	PS	7.5
MacKay (2021) [16]	US	2014–2017	15,224	PS	1.3
	G		3558		4.7
	NL		2795		3.6
	SWE		1406		2.7

Abbr.: PD = pancreatoduodenectomy, PS = pancreatic surgery, PDAC = pancreatic surgery of pancreatic ductal adenocarcinoma.

**Table 2:** Resection rates of PDAC in upfront resection or following NAT.

Author	*n*	UPF %	NAT %		
			R	BR	LA
Malleo (2025) [89]	681	90.5	96.1	86.6	61.9
Dickerson (2025) [90]	1194	72.6	80.6	-	-
Versteijne (2020) [74]	246	64 (R/BR)	52 (R/BR)		
Labori (2024) [77]	77	89 (R)	90 (R)		
Brown (2023) [75]	11,713	-	77.4	60.6	22.2
Truty (2021) [71]	254	-	-	90.1 (BR/LA)	
Ghaneh (2023) [91]	86	68 (BR)	-	58/55/50	-
Janssen (2019) [92]	313	-	-	67.8	-
Walma (2021) [93]	353	-	-	-	13

Abbr.: UPF = upfront resection, NAT = neoadjuvant therapy, R = resectable, BR = borderline resectable, LA = locally advanced.

## Data Availability

No new data were created or analyzed in this study. Data sharing is not applicable to this article.

## Abbreviations

ERAS	Enhanced Recovery After Surgery
CR-POPF	Clinically Relevant Postoperative Pancreatic Fistula
OS	Overall Survival
NAT	Neoadjuvant Therapy
